# Mitochondrial fission and stemness in prostate cancer

**DOI:** 10.18632/aging.102339

**Published:** 2019-09-29

**Authors:** Gianluca Civenni, Giuseppina M. Carbone, Carlo V. Catapano

**Affiliations:** 1 Tumor Biology and Experimental Therapeutics, Institute of Oncology Research (IOR) Università della Svizzera Italiana (USI), Bellinzona 6500, Switzerland

**Keywords:** mitochondrial dynamics, mitochondrial fission, cancer stem cells, prostate cancer, BRD4

Human cancers possess a hierarchical organization with cancer stem-like cells (CSCs) capable of both self-reproducing and generating a progeny of non-stem tumor cells with various degrees of differentiation and proliferative capacity [1]. CSCs contribute actively to intra-tumor heterogeneity, disease progression, metastatic spread and treatment failure [2]. In prostate cancer, CSCs, which are unaffected by standard androgen receptor (AR) targeting therapies, contribute to the emergence of aggressive and treatment resistant tumors [3]. To improve this scenario, new agents and treatment strategies are needed to eliminate CSCs along with bulk tumor cells.

In our study [4], we investigated the ability of inhibitors of bromodomain and extraterminal domain (BET) proteins to affect CSCs in prostate cancer models. BET proteins, like Bromodomain containing 4 (BRD4), recognize acetylated lysine residues on histone and non-histone proteins enabling the formation of transcription activating complexes at specific genomic sites [5]. BET protein inhibitors (BETi) block the formation of co-activator complexes and inhibit transcription in a cell-context specific manner [5]. BETi have anticancer activity and are currently evaluated in several clinical trials in many tumor types, including prostate cancer. However, whether BETi treatment affects prostate CSCs was not known.

To address this issue, we compared the effects of BETi and BRD4 knockdown in bulk tumor cells (BTCs) and prostate CSCs [4]. Surprisingly, we found the two cell subpopulations responded quite differently. BTCs experienced a transient growth arrest, which was more pronounced in AR-proficient cells (e.g., LNCaP cells) than in AR-negative cells (e.g., DU145). Conversely, BRD4 inhibition severely reduced tumor-sphere formation and self-renewal capacity at subsequent passages. Furthermore, BRD4 inhibition reduced tumor growth due to the consistent loss of tumor-initiating stem-like cells *in vivo*. These anti-CSC effects were seen both *in vitro* and *in vivo* equally in AR signaling proficient and deficient cells. Thus, inhibition of CSC expansion, rather than the transient arrest of BTCs, was driving the *in vivo* activity of BETi in the different xenograft models. Incidentally, these results emphasize the need of novel and diversified assays and experimental systems to fully assess the therapeutic potential of anticancer compounds. In our study, *in vitro*/*ex vivo* tumor-sphere assays and complementary tests unveiled new aspects of the activity of BETi and new potential applications in the clinical practice.

Next, we sought to determine the basis of the differential response of BTCs and CSCs to BRD4 inhibition. A revealing finding was the observation of differential susceptibility to induction of senescence [4]. Senescence is a form of progressive loss of replicative capacity linked to the aging of the cells ultimately leading to irreversible growth arrest. Senescence opposes the self-reproducing capacity of stem cells and is associated with progressive exhaustion of the stem cell pool [6]. In our prostate cancer models, we found that after BRD4 inhibition the fraction of senescent cells was considerably higher in CSC-enriched tumor-sphere cells compared to BTCs [4]. Thus, interfering with BRD4 caused senescence preferentially in prostate CSCs, which contributed to the exhaustion of the CSC pool.

What triggered senescence in prostate CSCs? The response to this question came by analyzing the transcriptional programs of BTCs and CSCs and the changes imposed by BRD4 inhibition [4]. First, we found significant differences in basal transcriptome of two cell subpopulations. Activated genes in tumor-sphere cells were preferentially associated with metabolic and mitochondrial processes, whereas genes expressed in BTCs were related mainly to cell proliferation. Second, only 25-35% of differentially expressed genes upon BETi treatment were down or upregulated in common between BTCs and CSCs. In line with the different basal transcriptomes, BETi repressed preferentially proliferation-related genes in BTCs and metabolism-related genes in CSCs.

**Figure 1:**
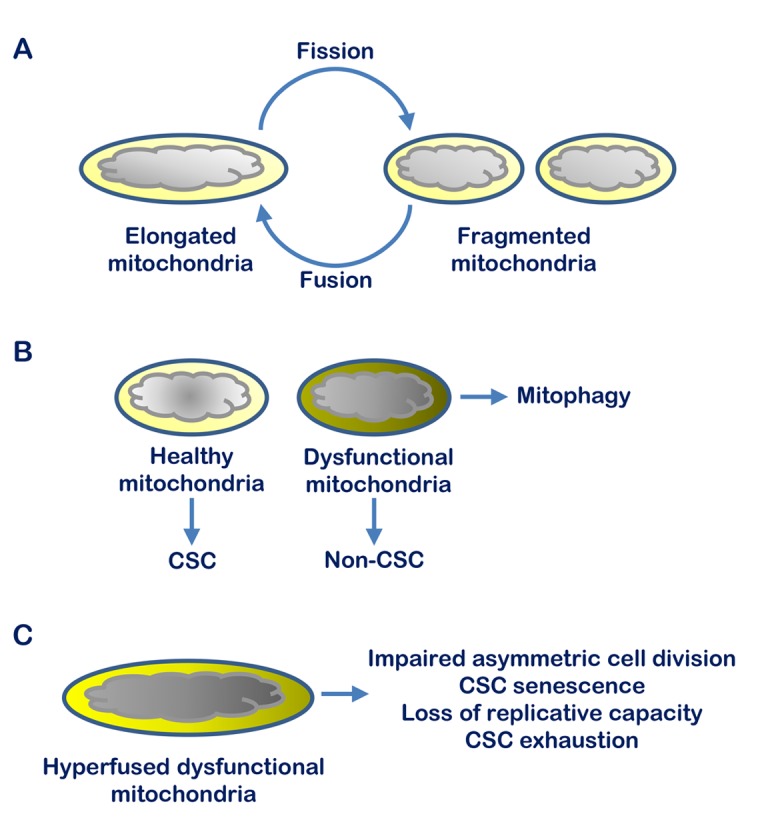
**Mitochondrial fission and maintenance of the cancer stem cell pool in prostate tumors.** (**A**) Mitochondrial dynamics comprises cycles of organelle fusion and fission ensuring the fitness of the mitochondrial population in a cell. (**B**) Mitochondrial fission allows removal of dysfunctional organelles by mitophagy and segregation of aged, non-functional mitochondria to non-stem cells during cell division. (**C**) Blocking mitochondrial fission, as in the case of BRD4 knockdown or treatment with BET inhibitors, impairs correct segregation and clearance of dysfunctional mitochondria during asymmetric cell division with consequent senescent and exhaustion of prostate cancer stem cells.

We further explored genes that could directly link the transcriptomic changes and the CSC-specific phenotypic effects. Among the genes preferentially upregulated in tumor-sphere cells and selectively repressed by BETi we identified *Mitochondrial Fission Factor* (*Mff*) as an interesting candidate [4]. MFF is the mitochondrial outer-membrane receptor of the Dynamin-related Protein 1 (DRP1) and a main driver of mitochondrial fission [7]. Enhanced mitochondrial biogenesis and dynamics are key elements in human cancers [7]. Mitochondrial dynamics, encompassing both mitochondrial fusion and fission, is critical for expansion and maintenance of normal and cancer stem cells determining the outcome of symmetric and asymmetric division and the balance between stem and non-stem cell progeny [7]. Mitochondrial fission enables proper partitioning of mitochondria between stem and non-stem daughter cells and altering this process prevents the correct execution of asymmetric cell division. As consequence, daughter stem cells inherit and accumulate dysfunctional mitochondria leading to senescence and irreversible loss of self-renewal capability [8]. Interestingly, we found that MFF was upregulated in hormone-refractory human prostate tumors and was overexpressed in CSCs compared to BTCs in multiple prostate cancer models [4]. Inhibiting BRD4 repressed *Mff* transcription in prostate CSCs causing the progressive accumulation of dysfunctional mitochondria and senescence. Importantly, *Mff* knockdown produced similar effects impairing mitochondrial segregation and promoting senescence, loss of proliferative potential and tumorigenic capability in prostate CSCs [4]. Furthermore, the phenotypic effects induced by BRD4 inhibition in tumor-sphere cells were fully rescued by ectopic expression of MFF. Because of the reduced dependency on mitochondrial fission, BTCs were less affected, showing no evidence of abnormal mitochondrial segregation and function or cell senescence after BRD4 and MFF depletion. Thus, mitochondrial dynamics represent a selective vulnerability that could be exploited to prevent the expansion and promote the exhaustion of prostate CSCs by failing asymmetric cell division and leading to symmetric commitment to differentiation and senescence (Figure 1).
